# Community structure of the solitary giant pandas is maintained by indirect social connections

**DOI:** 10.1186/s40462-022-00354-1

**Published:** 2022-12-01

**Authors:** Wenliang Zhou, Meng Wang, Yingjie Ma, Le Wang, Yibo Hu, Fuwen Wei, Yonggang Nie

**Affiliations:** 1 Key Laboratory of Animal Ecology and Conservation Biology, Institute of Zoology, Chinese Academy of Sciences, 1-5 Beichen Xilu, Chaoyang District, Beijing, China; 2 Center for Evolution and Conservation Biology, Southern Marine Science and Engineering Guangdong Laboratory (Guangzhou), Guangzhou, China; 3 University of Chinese Academy of Sciences, Beijing, China; 4 Center for Excellence in Animal Evolution and Genetics, Chinese Academy of Sciences, Kunming, China

**Keywords:** Giant panda, Social networks, Genetic network, Non-invasive genetic sampling, Social structure

## Abstract

**Background:**

Indirect interactions between individual solitary mammals, such as the giant panda, are often overlooked because of their nature, yet are important for maintaining the necessary sociality in solitary species.

**Methods and results:**

Here, we determined the genetic identity of all giant panda individuals in a local population and matched these identities with their associations to determine social network of this solitary animal. Total thirty-five giant panda individuals were found in our field survey, and we constructed genetic and social networks for thirty-three individuals who successfully obtained genetic, age and sex information. The results showed that sex had great impact on both social network and genetic network, and age may have the potential to influence the social network of the giant pandas. Adult males, mostly in the central of the social network, which appeared significantly larger network connections than adult females. Due to the female-biased dispersal pattern of wild giant pandas, male-male pairs showed higher relatedness than female-female ones and multi-generational patrilinear assemblages are expected in the study area.

**Conclusions:**

The relatedness of individuals has an influence on the formation of community social structure of giant pandas, and indirect interactions among solitary giant pandas potentially function to reduce competition for resources and inbreeding.

**Supplementary Information:**

The online version contains supplementary material available at 10.1186/s40462-022-00354-1.

## Background

One of the most important attributes of any animal population is its social structure, which embodies interactions among nearby conspecifics [1]. Multiple factors such as resource abundance, climate and environmental changes, and the reproductive status of individuals can affect the social structure of a species, causing fission and fusion events, and determining the frequency and cohesiveness of social interactions among individuals in a local population [2, 3]. Individual fitness is in part determined by social interactions among individuals, which further forms the basis of population growth and genetic diversity [4, 5].

Social network analysis enables the description of the frequency and patterns of social interactions among individuals and their social dynamics [6–9]. Most studies have focused upon social animals using direct and indirect behavioral observations [8–11]. However, indirect interactions are widespread among solitary species, which play an important role in maintaining their social structure [12]. For example, there is evidence that some solitary animals can exchange information through chemical communication without direct contact [13, 14]. Of 245 terrestrial carnivore species, 177 species are described as solitary and little is known about their social relationships [15]. Tracking animal movements in time and space can reveal potential indirect contact among solitary individuals [16].

The giant panda (*Ailuropoda melanoleuca*) is a solitary and non-cooperative bamboo eater species with a promiscuous mating system [17–20]. They rarely encounter each other, except for copulatory behavior during the mating season [21–23]. Telemetry data of wild panda movements indicates that they have highly overlapped home ranges but no direct contact during most of the year [18]. Accordingly, indirect interactions dominate their social behavior with loose social bonds, among different individuals and the potential social network in wild giant pandas. Giant pandas are highly dependent on chemical communication and can detect the presence of other individuals at great distances as well as information from pheromones deposited in the environment [22–24]. Genetic relatedness and kinship stabilise the structure of many animal social groups [25–28], such as spotted hyaenas (*Crocuta crocuta*, [29], African elephants (*Loxodonta africana*, [2]), giraffes (*Giraffa camelopardalis*, [30]) and dolphins (Tursiops spp., [31]). Previous molecular studies have confirmed that inbreeding avoidance in wild giant pandas is achieved passively through female dispersal rather than active mate choice [32–35]. Male offspring often established their home ranges adjacent to their mothers, while females generally disperse over long distances from their mothers’ home range once they are sexually mature [17, 18]. However, behavioral observations also indicate specific social structure in wild giant pandas [36]. Although several studies have confirmed the sex-dependent dispersal patterns, particularly for females and the social structure of wild giant pandas, how this dispersal behavior influences the socio-spatial organization of wild pandas remains unknown.

In brown bears, the philopatric behavior of females may result in a multi-generational cluster of related females, or “matrilinear assemblages” of successfully reproducing females [37]. Unlike other bears, giant pandas exhibit unique female-biased dispersal. Do giant pandas form multigenerational patrilinear assemblages as consequence of male philopatry? We address this question by using the spatial fecal sites to define individual indirect interactions of co-occurring giant pandas. We obtained the genetic identity of all individuals in the region and their respective associations from Foping National Nature Reserve. We then applied social network analysis to these wild giant pandas. Here we explore how female-biased dispersal influences the social interactions of solitary wild giant pandas, and if multigenerational patrilinear assemblages occur in this endangered solitary species.

## Methods

### Study area and study species

**Fig. 1:**
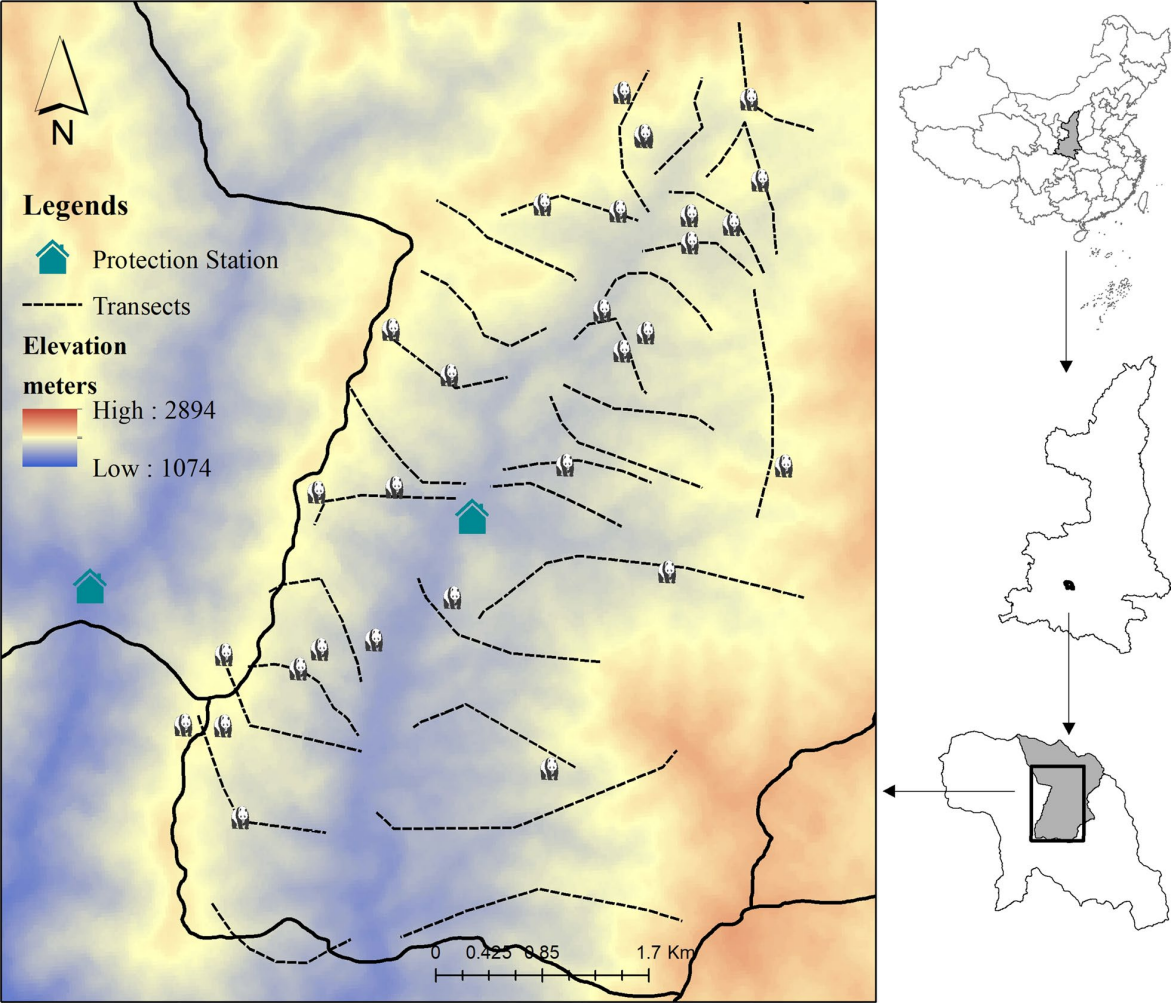
Foping National Nature Reserve, Shaanxi Province, China and the distribution pattern of 35 individuals in our study area. Each panda graphic symbol represents a identified individual, based on the fecal samples sites in our transect surveys

This study was conducted in the Sanguanmiao (SGM) protected station of the Foping National Nature Reserve (N 33° 32′–45′, E 107° 40′–55′) located in the Qinling Mountains of Shanxi Province, China (Fig. 1). This reserve was established primarily for the preservation of giant pandas. The recent third and fourth national surveys of the giant panda estimated that within the Foping reserve there are 70–80 wild individuals; the largest known population density [38, 39].

Two bamboo species, the wood bamboo (*Bashania fargesii*) and arrow bamboo (*Fargesia qinlingensis*) comprise most of diet of the giant panda in the low (below 1900 m a.s.l) and high (above 1900 m a.s.l) habitat, respectively [40]. In the study area, foraging giant pandas track the seasonal phenology of the two bamboo species [19, 20, 41]. For almost nine months, from late August to early June, pandas feed on wood bamboo leaves and shoots at lower altitude, and move to higher altitude to use arrow bamboo in the summer for 3 months [17, 41]. Here, we focus on the nine-month period when giant pandas are in lower habitat because the home range is relatively stable during this time [42].

### Noninvasive genetic sampling and field observations

To investigate the social network and socio-spatial organization of wild giant pandas, we collected genetic information from all individuals in our study area. From November 2017 to April 2018, we performed an exhaustive noninvasive genetic sampling of panda feces encountered in the study area. Thirty transects dispersed throughout the study area were established, and we systematically surveyed these transects every month to collect the fresh fecal samples for genetic analysis. A comprehensive, uniform search was done in the survey area by default. In the field, 10 g of feces was peeled from the outer layer and stored at −20 °C at the field base, and then transported in ice to the lab for the following age and sex and individual analysis at the Institute of Zoology, CAS. All the locations of the fecal samples were recorded by GPS.

With the approval of the State Forestry Administration of China, four giant pandas (2 males and 2 females) were fitted with high frequency GPS/VHF radio collars (Lotek Wireless Inc., Ontario, Canada). We used the GPS collars to locate and record behavioral interactions among the collared pandas and with other individuals (e.g., aggression and breeding). From these data we determined territory size and derived the social relationships and structure of the local panda population.

### Age determination

The fecal fragment size was used as a key indicator of the age-size estimation of giant pandas. This method has been applied successfully in the national surveys of the giant panda [38, 39]. We measured the length and width of bamboo leaf fragments randomly, and no less than forty fragments were measured in each fecal sample. These measurements were compared to leaf fragments from the feces of six giant pandas (XiYue,DianDian; ZhenZhen; HuZi; NiuNiu and LanNi) whose ages were known as controls for the age estimations (Table 1). Based on the average length and width of bamboo leaf fragments in feces, all individuals were categorized into one of two main age classes: sub-adults (less than 5.5 years old) and adults (older than 5.5 years) [43]. The sub-adults including the cubs, who are not yet weaned (0–1.5 years) either do not eat bamboo or chewed it too finely. Thus, no fecal fragment size data was collected for cubs. By using the length and condition of the leaf fragment, we were able to identify some elderly pandas, whose ability to chew bamboo leaves because of tooth wear had declined resulting in significantly larger leaf fragments.

**Table1:** Age class information of giant pandas in SGM

Individual number (name)	Sex	Average length (mm) (Mean ± SE)	Average width (mm) (Mean ± SE)	Age class (Age estimated)
S1 (XiYue)	Male	33.181 ± 0.714	8.170 ± 0.183	Adults (17 years)
S2 (DianDian)	Male	33.523 ± 0.590	8.083 ± 0.235	Adults (17 years)
S3 (ZhenZhen)	Female	25.276 ± 0.561	6.764 ± 0.240	Adults (14 years)
S4 (HuZi)	Male	23.462 ± 0.660	8.890 ± 0.445	Adults (7 years)
S5 (NiuNiu)	Female	29.141 ± 0.482	7.971 ± 0.338	Adults (16 years)
S8 (HouBao)	Male	–	–	Sub-adults
S9 (DG)	Male	27.362 ± 0.583	7.680 ± 0.303	Adults
S10 (DongDong)	Female	29.205 ± 0.644	8.541 ± 0.222	Adults
S11 (ZLC)	Female	34.115 ± 0.562	9.201 ± 0.229	Adults
S12 (LiLi)	Female	22.879 ± 0.411	7.442 ± 0.172	Sub-adults
S13 (DZC)	Female	26.775 ± 0.551	5.768 ± 0.231	Adults
S14 (LJG)	Male	26.339 ± 0.461	7.880 ± 0.271	Adults
S15 (DZC)	Male	26.254 ± 0.494	7.675 ± 0.303	Adults
S16 (Jiang)	Female	43.573 ± 0.468	13.350 ± 0.483	Adults
S17 (GuGu)	Male	32.428 ± 0.385	9.213 ± 0.305	Adults
S18 (HJY)	Male	27.795 ± 0.631	5.590 ± 0.295	Adults
S19 (ZWG)	Male	23.414 ± 0.782	11.015 ± 0.430	Adults
S20 (ZW)	Female	28.790 ± 0.399	7.421 ± 0.412	Adults
S21 (DLZG)	Male	39.668 ± 0.795	12.911 ± 0.444	Adults
S22 (WFG)	Male	22.090 ± 0.529	8.122 ± 0.309	Sub-adults
S23 (XLZG)	Female	27.956 ± 0.516	7.581 ± 0.380	Adults
S24 (XLZG)	Male	–	–	Sub-adults
S25 (XMDG)	Female	23.068 ± 0.442	6.834 ± 0.368	Adults
S26 (HNB)	Male	18.666 ± 0.366	6.089 ± 0.187	Sub-adults
S27 (ZWG)	Female	30.027 ± 0.330	6.874 ± 0.166	Adults
S28 (XYP)	Female	28.019 ± 0.457	7.109 ± 0.203	Adults
S29 (ZLC)	Female	23.861 ± 0.511	7.308 ± 0.196	Adults
S30 (JJG)	Female	21.270 ± 0.470	7.601 ± 0.216	Sub-adults
S31 (ZWG)	Female	–	–	Adults
S32 (LJG)	Female	–	–	Adults
S33 (LZC)	Female	–	–	Adults
S34 (LanNi)	Female	23.732 ± 0.389	7.488 ± 0.196	Adults (10 years)
S35 (DongYang)	Male	29.434 ± 0.695	9.770 ± 0.328	Adults
S36 (XiaXia)	Unknown	–	–	Sub-adults
S37 (XiaoYang)	Unknown	–	–	Sub-adults

### Sex and individual identification

We extracted total DNA from fecal samples using the QIAamp DNA Stool Mini kit (QIAGEN, Hilden, Germany) following the manufacturer’s instructions. Fifteen microsatellite primers Ame-μ5, μ10, μ11, μ13, μ15, μ22, μ24, μ26, μ27, Aime1, AY79, AY95, AY161213, AY217, GP7 [32, 44, 45] were used to amplify DNA extracts from fecal samples. A multi-tube amplification approach [46] was used and we amplified each extract three times. If the genotype could not be determined, we performed two additional amplifications until we obtained reliable genotypes. PCR amplifications followed the method of previous studies [32]. PCR products were separated using an ABI 3730xl sequencer and scored using GeneMarker® v 2.2.0 (SoftGenetics LLC). After obtaining multi-locus combined genotypes of each sample, we performed individual identification following Zhan et al. [47]. Micro-Checker [48] was applied to detect the presence of genotyping errors such as null alleles, large allele dropout or stuttering. Mstools plug-in in Microsoft Excel was used to find the matching genotypes in genotyping data.

Two species-specific sexing primer pairs ZX1 (210 bp) and ZF (130 bp) were designed for sex determination [47], conducted three times for each DNA extract. A sample was identified as male if at least two experiments showed two bands (210 bp ZX1 and 130 bp ZF band), and as female if only one band (130 bp) was produced. Blood DNA from captive male and female giant pandas was used as positive controls, and a negative control without a DNA extract, were amplified in each PCR.

### Delineation of social partners and networks

Previous studies have shown that the average area of pandas’ winter habitat is no less than 6.02 km^2^ in Qinling Mountains [17, 19, 20]. From late August to early June, pandas forage wood bamboo leaves and shoots at winter habitat in SGM study area (less than 20 km^2^, Fig. 1), where maintains the highest population density during the third and fourth national survey [38, 39]. Therefore, it is easy to observe that a large number of giant pandas with developed olfactory communication systems [22, 23] gather together in order to share the limited winter habitat [19, 20, 41]. Meanwhile, the evidence from the collar data showed that, monitored individuals could fully use the winter habitat in SGM study area with no difference in preference [42]. Hence, there exist great possibility that complex direct or indirect social connections between them to maintain the stability of the high-density population community. Here, we hypothesized that all pandas in SGM study area a community unit and defined the existence of social associations between all individuals who was successfully identified in each month survey, whether or not their direct contacts were observed. The association records were binary. In other words, we interpret each survey as a thorough scan of the study area, and all individuals found in the survey are considered to have associations with a value of "1", while the remaining individuals not found in this survey are given a value of "0", which is considered to have no associations.

Therefore, we adopted a null model based social network analysis frame to describe the association dynamics between the individuals in SGM study area [49]. The null model enables us to test if the specific social structure exists, and explicitly separate out alternative hypotheses. We used node permutation test and the permutations were run for 1000 times for each model. The two-tailed values were used and the observed test statistic could be either greater or smaller than random [49]. Sex and age were also tested if they have impacts on the structure the social network. Nodes in the social network represent pandas. The edges represent associations between giant panda pairs, and the weight of an edge represent how frequently they associate [50].

### Estimation of relatedness and genetic network 

We used KINGROUP v2 [51] to estimate pairwise genetic relatedness between pairs of individuals, based on the allele frequencies calculated from all pandas identified in this study [52]. Theoretically, relatedness values (R) range from −1 to 1, and first-class relatives (e.g., parent–offspring, full-sibs), second-class relatives (e.g., grandparent–grandchild, half-sibs) and third-class relatives (e.g., first-cousin) are 0.5, 0.25 and 0.125, respectively. If relatedness values (R) are less than 0.125, we defined no relatedness. The size of R value represents the strength of genetic relationship at different levels, which is endowed with the thickness of lines between nodes in the genetic network. We adopted the null-model method to test the genetic social network among the identified individuals. Similarly, sex and age were tested that if they have impact on the genetic network structure, and the permutations were run for 1000 times. Then, we estimated the male-male (hereafter MM), male–female (hereafter MF) and female-female (hereafter FF) pairwise relatedness of all pandas in this study.

### Statistical analysis

We also adopted Wilcoxon rank sum test to compare the relatedness differences between MM and FF pairs of all the adults. We compared the genetic network and the social network using Mantel test (Spearman correlation) to test the strength of social associations and the genetic relatedness. We used R package “asnipe” [53] to do the social and genetic network permutation analysis and used “igraph” [54] to calculate node properties of degree centrality for both networks following the method of Farine [49]. Degree centrality is measured by the degree of nodes, and higher degree of nodes means the higher degree centrality. The social and genetic networks of all individuals in the SGM study area were also drawn by “igraph”. R statistical software (ver. 3.5.2) was used to analyze all the data [55]. The data are represented as mean ± SEMs.

## Results

### Population size of giant pandas in our study area

A total of 346 fresh giant panda fecal samples were collected, of these 221 samples were successfully genotyped, representing thirty-three unique giant panda individuals including 15 males and 18 females. Together with two new born cubs that we observed during this study, we identified thirty-five individuals in the SMG study area. The age determination indicated that there were twenty-seven adults (including three elderly pandas) and eight sub-adults (including four cubs) in our study area (Table 1). The two new cubs rarely defecated and sex determination and microsatellite genotyping were not possible for them.

### Social networks of giant pandas

**Fig. 2:**
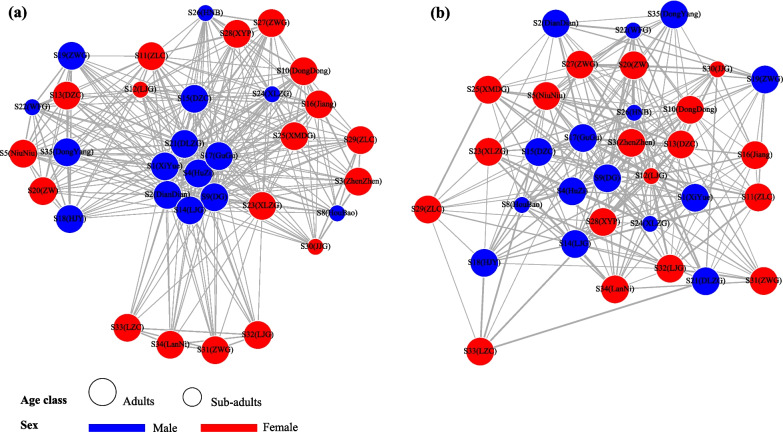
(**a**) The social network of wild pandas in SGM study area, (**b**) The genetic network of wild pandas in SGM study area. The nodes represent panda individuals and the thickness of the lines between nodes is proportionate to the degree of social connection strength and relatedness between those individuals

**Fig. 3:**
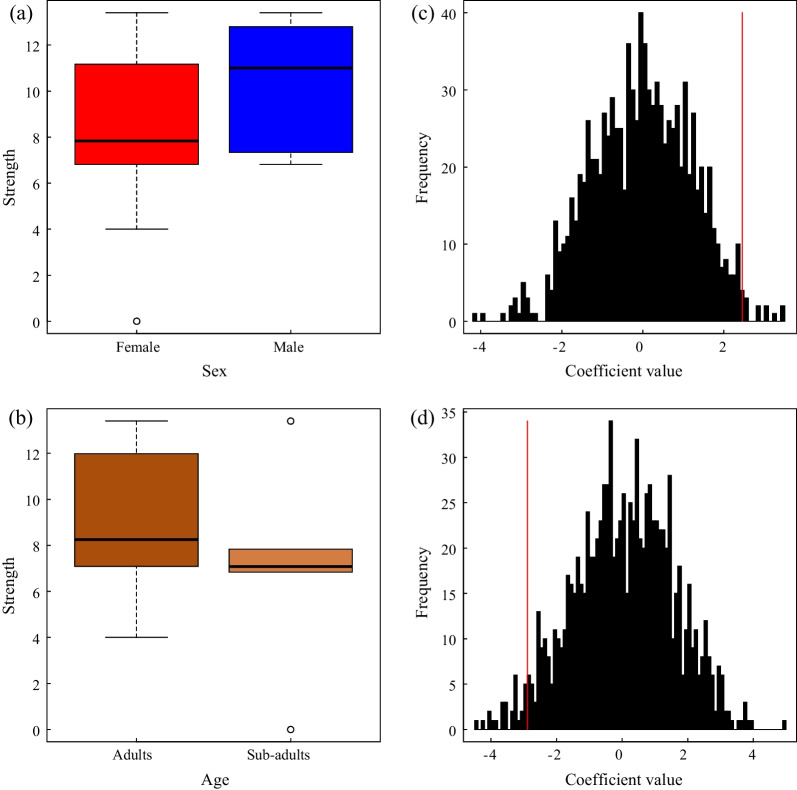
The impact of (**a**) sex and (**b**) age on wild panda social network. The distribution of permuted model estimates for the effect of (**c**) sex, (**d**) age. The redlines indicate the observed value of the coefficients

A total of thirty-five pandas were found in the SGM study area, which covers an area of less than 20 km^2^, indicating a very high population density and a high degree of overlap between individuals. In order to analyze the influence of sex and age on the community, we removed two new cubs (S36 (XiaXia) and S37 (XiaoYang)) who failed to identify their sex when constructing the social network. Then, we total recorded 336 social associations by the 33 identified individuals (Fig. 2a, Additional file 1: Table S1), and the connectance index was only 0.636 in the social network of SGM population. Seven individuals have a highest degree centrality including four males and three females (Additional file 1: Table S2), and the average degree of each individual was 20.363 ± 7.219 (Additional file 1: Table S1). Male pandas generally associate more individuals, which made them in the central of the social network (Fig. 2a). The sex of giant pandas in the community had a significant effect on the giant panda social network, and males (0.489 ± 1.397) have a higher strength (weighted degree) than females (−0.847 ± 1.830) (Fig. 3a, b, *p* = 0.022). The effect of age was not significant at 5% level. Consulting the extremely low encounter rate of sub-adult in the wild [39], we consider that age could potentially influence the social network, which may need more proof in the future (Fig. 3c, d, *p* = 0.056).

### Relatedness of all pandas in study area

**Fig. 4:**
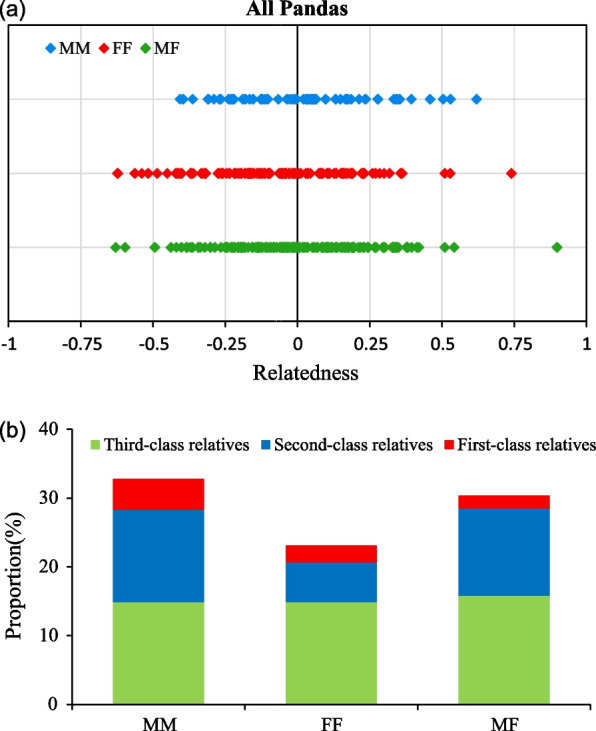
(**a**) The genetic relatedness distribution of MM, MF and FF pairs of all individuals, (**b**) The proportion of different class relatives on MM, MF and FF pairs

To measure kin social relations, we estimated the relatedness values (R) between same sex pairs: MM pairs, FF pairs and different sex pairs: MF pairs. The estimated relatedness (R) between all same and different sex pairs (n = 342) ranged from −0.633 to 0.897 (Fig. 4a). MM pairs (n = 66) were significantly more closely related than FF pairs (n = 119) (Wilcoxon rank sum test: *W* = 3108.5, *p* = 0.015). For all individuals, 32.84% of the MM pairs were relatives (R ≥ 0.125), which was higher than MF pairs (30.38%) and FF pairs (23.14%) (Fig. 4b). In MM pairs, first-class relatives were 4.47%, second-class relatives 13.43% and third-class relatives 14.93%. In contrast, 2.48% of the FF pairs were first-class relatives, 5.79% second-class relatives and 14.88% third-class relatives (Fig. 4b). FF pairs were notable for fewer second-class relatives and first-class relatives than MM pairs. The MF pairs comprised 1.90% first-class relatives, 12.66% second-class relatives and 15.82% third-class relatives (Fig. 4b). In conclusion, MM pairs showed highest kinship and first-class relatives than MF pairs and FF pairs, which confirmed the clustering of male relatives of panda community in the study area to a certain extent.

### Genetic networks of giant pandas

**Fig. 5:**
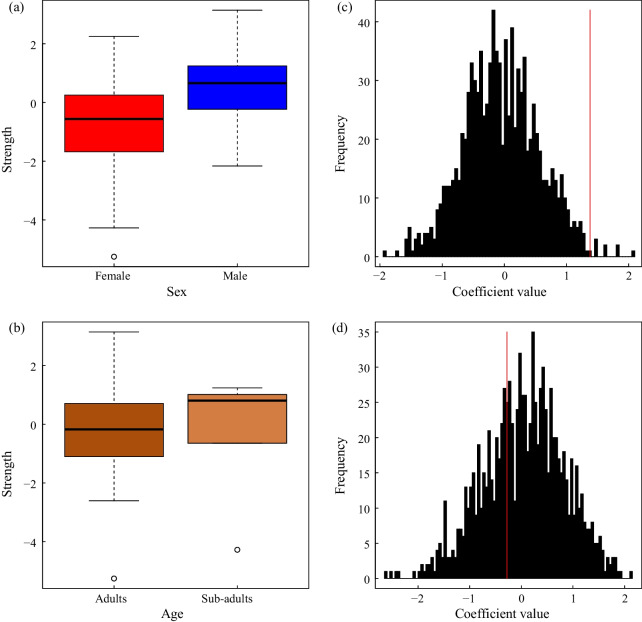
The impact of (**a**) sex and (**b**) age on genetic network of wild pandas. The distribution of permuted model estimates for the effect of (**c**) sex, (**d**) age. The redlines indicate the observed value of the coefficients.

The genetic network was based on three classes of relatives among all individuals in the community, with 33 vertices (two cubs have no genetic information) and 179 edges (Fig. 2b, Additional file 1: Table S1). The connectance index and average degree in genetic network were 0.269 and 9.676 ± 0.851, which is lower than in the social network (Additional file 1: Table S1). Male individuals are at the center of the network in the social network, the female giant panda also shows high degree centrality in the genetic network (Additional file 1: Table S3). Among them, the three pandas located in the central of the genetic network and have the highest degree centrality were S17 (GuGu), S12 (LiLi) and S13 (DZC). Except for S17 (GuGu), who is a male, the other two are adult females. The results showed that males (0.489 ± 1.397) have a higher strength (weighted degree) than females (−0.847 ± 1.830) on genetic network (*p* = 0.004, Fig. 5a, c), but adults and sub-adults showed no difference in strength (*p* = 0.330, Fig. 5b, d). The result of the mantel test showed that giant panda’s social network was significantly associated with genetic network (Number of permutations: 1000, *p* = 0.043).

## Discussion

Despite few direct contact events, strong community social structure exists in wild giant pandas, commensurate with the findings of a previous study [36]. Giant pandas appear to possess a kinship bonded society in spite of their solitary nature most of the time. As observed in giant pandas, male-biased post-natal dispersal is most common mode of dispersal among mammal species and males disperse greater distances than females [56, 57]. For these species, females often form stronger associations (female-bonded) with kin than non-kin [31, 58, 59], and the clustering of kin, facilitated by female philopatry, can result in significantly higher within-community relatedness levels than expected by random assortment [60], which may lead to inbreeding. However, several previous studies indicate that the primary mechanism of inbreeding avoidance in giant pandas may be passive female-biased natal dispersal [32]. Our research supports this female-biased natal dispersal pattern in wild giant pandas as (1) males were more closely related to each other than females, and (2) female dispersal and male philopatry in the SGM study area. The proportion of relatives in MM pairs was significantly higher than in MF and FF pairs of all individuals. Multigenerational patrilinear assemblages are apparent in the SGM study area and the relatedness of individuals has an influence on the formation of community social structure of giant pandas.

Social interactions by adult males occurred more frequently than measured for adult females, indicating that males interact more frequently with their neighbors than females do. Female giant pandas appear to keep social contact with other individuals to a minimum. For females, home range sizes are often determined by the availability of nutritional resources essential to maintaining themselves and their cubs [61, 62]. Bamboo that supports the nutritional needs of the giant panda is abundant at high-density in the study area [19, 20, 41] allowing adult females small home range sizes and minimal encounters with other individuals [63]. In contrast, access to mates may be the main determinant of territory size for males, not habitat quality and food availability [64]. Adult male panda home ranges encompass the home ranges of a number of potential female mates, a phenomenon observed where sexual selection is driven by female mate choice [15, 61, 62]. Male pandas not only extended their home range sizes with frequent scent-marking on the home range boundary [22], but also actively seek contact with potential female mates to increase their mating opportunities.

The patrilinear social structure in wild pandas may provide additional benefits including reduced competition among female kin for resources and inbreeding avoidance through female dispersal [65]. Male giant pandas usually established home ranges near their mothers’, which has been confirmed in a previous study [17]. One of the pairs was S3 (Zhen Zhen) and her son S4 (Hu Zi) born in September 2011, which was confirmed by our long-term collar monitoring. Previous studies have demonstrated that giant pandas can recognize their cubs [66, 67]. The territory of sub-adult males was close to their mother and cousins, which could minimize competition for food resources and the risk of infanticide in the long-run. In addition to the doubtless mother–child relationship, MF pairs showed a lowest degree of first-class relatives, resulting in a low likelihood inbreeding in our study area.

Nevertheless, the genetic approach is a useful method for determining the social structure of the giant panda. Our study suggests that the community social structure of giant pandas may influenced by kinship and indirect interactions play a key role in maintaining social structure. These findings complement current knowledge of the social structure of wild giant pandas, especially the importance of indirect interactions between individuals, which has been ignored in previous studies [17, 18]. These revelations challenge us to reconsider how social behaviors, including direct and indirect social interactions, influence the distribution, territoriality, intraspecific competition and inbreeding avoidance of wild giant pandas.

## Conclusions

Indirect interactions are important components of social behavior in many solitary species that often use chemical signals to exchange information. However, it is difficult to detect the influence of indirect interaction with traditional methods (e.g. field behavior observation). In this study, we firstly constructed the social network and genetic relatedness network on a solitary species to explore how female-biased dispersal influences the social interactions of solitary wild giant pandas. The integration of social network techniques with classical non-invasive genetic analysis marks a new advance in understanding the social spatial structure of solitary species. Monitoring and quantifying these indirect interactions not assist our understanding of sexual selection, reproduction, intra-specific competition, and the social spatial organization of solitary species, but help to controlling the spread of parasites and infectious diseases in wildlife.

## Supplementary Information

**Additional file 1. Table S1** Comparison of social network and genetic network parameters. **Table S2** The social network index of all individuals in SGM study area. **Table S3** The genetic network index of all individuals in SGM study area.

## Data Availability

Please contact authors for data requests.
