# Supplementary material for: Community structure of the solitary giant pandas is maintained by indirect social connections
Source: Mov Ecol. 2022 Dec 1;10:53. doi: 10.1186/s40462-022-00354-1 (PMC9716724; doi:10.1186/s40462-022-00354-1)
Supplement: Supplementary file 1 — Additional file 1. Table S1 Comparison of social network and genetic network parameters. Table S2 The social network index of all individuals in SGM study area. Table S3 The genetic network index of all individuals in SGM study area. [file 40462_2022_354_MOESM1_ESM.docx]

**Additional file 1 of**

**Community structure of a solitary mammal species with covert social connections**

Wenliang Zhou^1,2,†^, Meng Wang^1,3,†^, Yingjie Ma^1,3^, Le Wang^1,3^, Yibo Hu^1,3,4^, Fuwen Wei^1,2,3,4^, Yonggang Nie^1,3,4*^

^†^Wenliang Zhou and Meng Wang contributed equally to the manuscript

Correspondence: Yonggang Nie ; Email: nieyg@ioz.ac.cn

^1^ Key Laboratory of Animal Ecology and Conservation Biology, Institute of Zoology, Chinese Academy of Sciences, Beijing, China.

^2^Center for Evolution and Conservation Biology, Southern Marine Science and Engineering Guangdong Laboratory (Guangzhou), Guangzhou, China.

^3^ University of Chinese Academy of Sciences, Beijing, China.

^4^ Center for Excellence in Animal Evolution and Genetics, Chinese Academy of Sciences, Kunming, China.

**This file includes:**

**Additional file 1: Table S1-S3**

**Additional file 1: Table S1** Comparison of social network and genetic network parameters.

| **Network Index** | **Social network** | **Genetic network** |
| --- | --- | --- |
| Number of edges | 336 | 179 |
| Number of vertices | 33 | 33 |
| Connectance | 0.636 | 0.269 |
| Average degree | 20.363 | 9.676 |
| Average path length | 0.407 | 1.896 |
| Diameter | 0.7 | 0.781 |
| Edge connectivity | 11 | 2 |
| Clustering coefficient | 0.768 | 0.458 |
| No clusters | 1 | 1 |

**Additional file 1: Table S2** The social network index of all individuals in SGM study area.

| **Individual number (name)** | **Sex** | **Degree centrality** |
| --- | --- | --- |
| **S1(XiYue)** | Male | **32** |
| S2(DianDian) | Male | 21 |
| S3(ZhenZhen) | Female | 24 |
| S4(HuZi) | Male | 24 |
| S5(NiuNiu) | Female | 16 |
| **S8(HouBao)** | Male | **32** |
| S9(DG) | Male | 28 |
| S10(DongDong) | Female | 21 |
| **S11(ZLC)** | Female | **32** |
| S12(LiLi) | Female | 16 |
| S13(DZC) | Female | 16 |
| **S14(LJG)** | Male | **32** |
| S15(DZC) | Male | 16 |
| **S16(Jiang)** | Female | **32** |
| S17(GuGu) | Male | 16 |
| S18(HJY) | Male | 21 |
| S19(ZWG) | Male | 21 |
| S20(ZW) | Female | 21 |
| S21(DLZG) | Male | 17 |
| S22(WFG) | Male | 17 |
| S23(XLZG) | Female | 17 |
| S24(XLZG) | Male | 15 |
| S25(XMDG) | Female | 15 |
| S26(HNB) | Male | 15 |
| S27(ZWG) | Female | 11 |
| S28(XYP) | Female | 11 |
| S29(ZLC) | Female | 11 |
| S30(JJG) | Female | 11 |
| S31(ZWG) | Female | 16 |
| **S32(LJG)** | Female | **32** |
| S33(LZC) | Female | 16 |
| S34(LanNi) | Female | 15 |
| **S35(DongYang)** | Male | **32** |
| S36(XiaXia) | Unknown | - |
| S37(XiaoYang) | Unknown | - |

**Additional file 1: Table S3** The genetic network index of all individuals in SGM study area.

| **Individual number (name)** | **Sex** | **Degree centrality** |
| --- | --- | --- |
| S1(XiYue) | Male | 15 |
| S2(DianDian) | Male | 10 |
| S3(ZhenZhen) | Female | 16 |
| S4(HuZi) | Male | 15 |
| S5(NiuNiu) | Female | 7 |
| S8(HouBao) | Male | 12 |
| S9(DG) | Male | 13 |
| S10(DongDong) | Female | 5 |
| S11(ZLC) | Female | 4 |
| **S12(LiLi)** | Female | **19** |
| **S13(DZC)** | Female | **19** |
| S14(LJG) | Male | 13 |
| S15(DZC) | Male | 8 |
| S16(Jiang) | Female | 9 |
| **S17(GuGu)** | Male | **20** |
| S18(HJY) | Male | 11 |
| S19(ZWG) | Male | 2 |
| S20(ZW) | Female | 11 |
| S21(DLZG) | Male | 4 |
| S22(WFG) | Male | 10 |
| S23(XLZG) | Female | 5 |
| S24(XLZG) | Male | 12 |
| S25(XMDG) | Female | 9 |
| S26(HNB) | Male | 7 |
| S27(ZWG) | Female | 6 |
| S28(XYP) | Female | 15 |
| S29(ZLC) | Female | 7 |
| S30(JJG) | Female | 4 |
| S31(ZWG) | Female | 6 |
| S32(LJG) | Female | 10 |
| S33(LZC) | Female | 3 |
| S34(LanNi) | Female | 12 |
| S35(DongYang) | Male | 10 |
| S36(XiaXia) | Unknown | - |
| S37(XiaoYang) | Unknown | - |
